# SiaRNA: a siamese neural network with bidirectional cross-attention for pairwise siRNA-mRNA efficacy prediction

**DOI:** 10.3389/fbinf.2026.1827877

**Published:** 2026-08-05

**Authors:** Vaishnavi Sapireddy, Rajkumar Nathi, Venkata Harshit Meruva, Varun Raju Nannapuraju, Bhargava Chary Basangari, Vani Kondaparthi

**Affiliations:** 1 Department of Computer Science, Neil Gogte Institute of Technology, Hyderabad, Telangana, India; 2 Drugparadigm Research Lab, Hyderabad, Telangana, India; 3 Department of Computer Science and Engineering (AI&ML), Keshav Memorial Engineering College, Hyderabad, Telangana, India; 4 Department of Computer Science and Engineering, Keshav Memorial Engineering College, Hyderabad, Telangana, India; 5 Department of Chemistry, Keshav Memorial Engineering College, Hyderabad, Telangana, India

**Keywords:** cross-attention mechanism, deep learning, pairwise interaction modelling, post-transcriptional gene silencing, RNA interference (RNAi), siamese neural network, siRNA efficacy prediction

## Abstract

**Introduction:**

Small interfering RNA (siRNA) therapeutics have extraordinary potential for targeted gene silencing. They mediate post-transcriptional gene regulation by binding to complementary messenger RNA (mRNA) sequences and degrading them, thereby preventing the production of unwanted proteins. Recent machine learning and deep learning frameworks for predicting siRNA efficacy have only achieved moderate success as these models solely rely either on handcrafted features or on sequential relations and therefore cannot capture the full complexity of siRNA-mRNA interactions.

**Methods:**

In the above context, we propose SiaRNA, which uses a Siamese Neural Network for feature-derived representations and a bidirectional cross-attention mechanism for sequence-level relationships. It uniquely identifies mRNAs and their corresponding siRNAs as paired entities, allowing unified and context-aware modeling. Unlike previous models, which discard 2-nucleotide (2-nt) overhangs at the 3′ end while using 21-nt efficacy labels, SiaRNA both trains and tests on 21-nt sequences to ensure biologically consistent predictions.

**Results:**

Our model sets a new performance benchmark, outperforming previous state-of-the-art models. SiaRNA is trained on the HUVK dataset, achieving an accuracy of 0.881, and its generalization is confirmed by testing on the independent Simone dataset.

**Discussion:**

The results prove SiaRNA’s potential as a reliable and biologically accurate framework to guide siRNA design and improve therapeutic outcomes. On performing a case study using Patisiran siRNA and its target transthyretin (TTR) mRNA, an efficacy value of 0.7134 was observed indicating that our model can successfully identify therapeutically effective targets.

## Graphical Abstract

**Figure:**
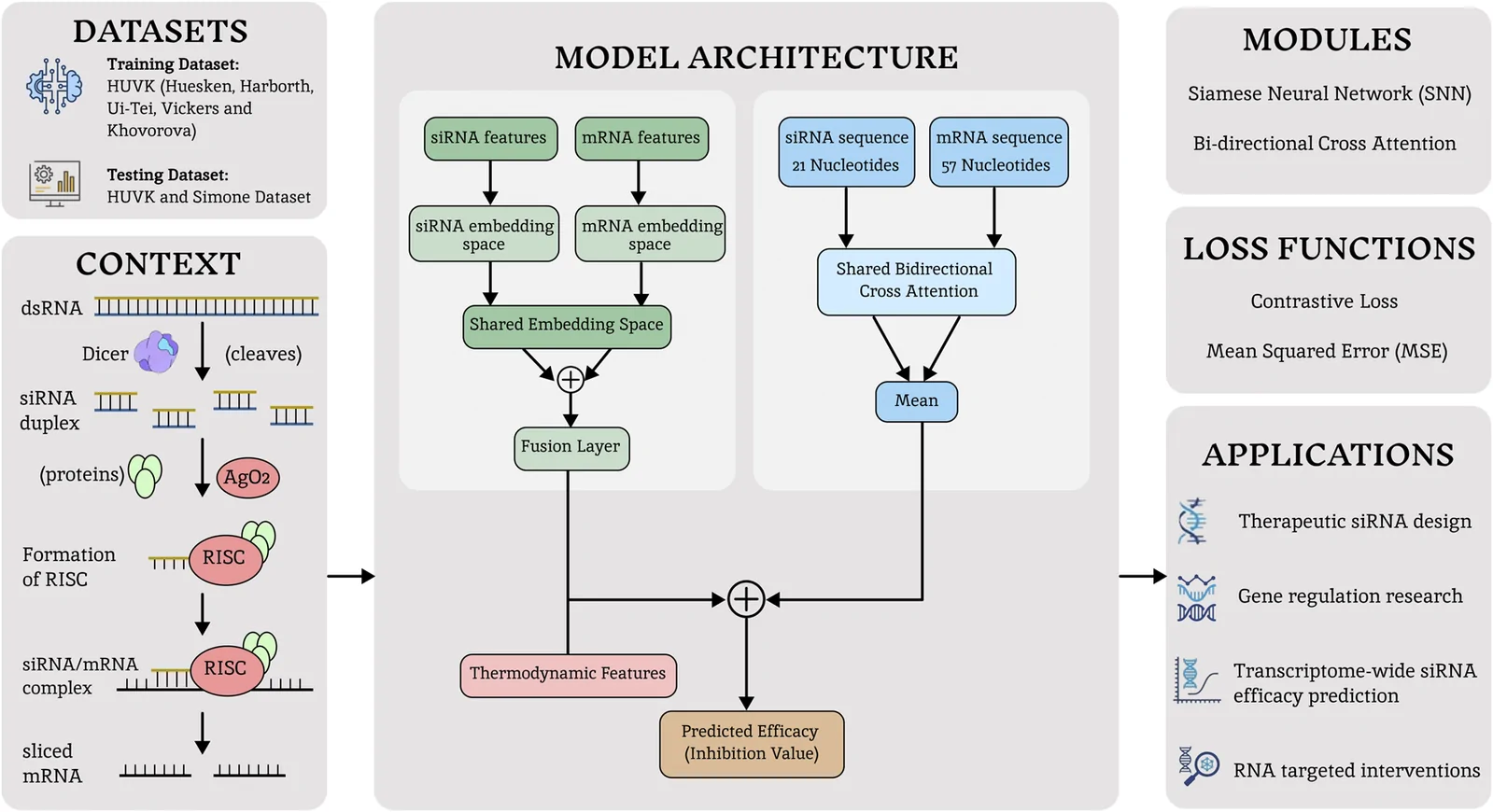

## 1 Introduction

RNA interference (RNAi) (Hannon, 2002) is a natural cellular defense mechanism found across eukaryotic organisms that regulates gene expression at post-transcriptional level. Transcription is the first step in gene expression, where genetic information encoded in DNA is copied into messenger RNA (mRNA) within the cell nucleus by RNA polymerase enzymes. The newly synthesized mRNA molecule then exits the nucleus and enters the cytoplasm, where translation occurs, during which the ribosomes decode the mRNA to synthesize the corresponding proteins.

Proteins are essential molecules in the body, responsible for carrying out almost every cellular function. However, protein production can be disrupted due to genetic mutations, transcription or translation errors (Yuan et al., 2024) and viral infections (Rozman et al., 2023), leading to serious conditions such as cancer (Hashemi et al., 2023), genetic disorder, metabolic disorder and neurodegenerative diseases. To control such abnormalities, cells employ the RNA interference (RNAi) pathway, which regulates protein production through double-stranded RNA (dsRNA). dsRNA is a type of RNA that can naturally form within the cells, such as during viral infections. Inside the cell, the long double-stranded RNA (dsRNA) is recognized by an enzyme called Dicer, a special type of ribonuclease (RNase). Dicer cleaves the dsRNA into smaller fragments known as small interfering RNAs (siRNAs) (Wilson and Doudna, 2013), typically 19–23 nucleotides in length. To induce RNAi, small interfering RNAs (siRNAs) or short hairpin RNAs (shRNAs) are introduced into the cell, where they mimic the natural products of the RNAi pathway. These siRNAs are then incorporated into the RNA-induced silencing complex (RISC), which is a multi-protein effector complex that carries out gene silencing. Inside RISC, the siRNA loses one of its strands, called passenger strand, while the other strand, guide strand, is retained. The guide strand serves as a template to find complementary mRNA. By means of base-pairing interactions, it scans the cellular mRNA to identify target sequences. Once the siRNA-mRNA duplex forms, Argonaute 2 (AGO2) protein, which is the catalytic core of RISC, cleaves the target mRNA at the site of the complementary region. The resulting mRNA fragments are then degraded by RNA-degrading enzymes that prevent the translation of mRNA into protein, thus silencing the target gene. This selective process makes sure that only specific genes are silenced, allowing the cell to accurately control the synthesis of proteins and maintain cellular balance.

Advancements in computational biology and machine learning have helped grow the study of RNA interference beyond traditional experimental methods. Various state-of-the-art computational models have been developed for the prediction and analysis of siRNA-mRNA interactions like s-Biopredsi (Huesken et al., 2005), DSIR (Filhol et al., 2012) and i-Score (Ichihara et al., 2007). These developed models for siRNA efficacy prediction span rule-based frameworks to neural network architectures and advanced data mining techniques. Despite the progress made with the help of these frameworks, there are still many persistent challenges such as inconsistency in performance across datasets due to variations in experimental procedures, high dependency on feature selection and an inability to model non-linear interactions.

To address these limitations, Convolution Neural Network (CNN) (Han et al., 2018) based models were employed to analyze sequence context along with thermodynamic properties which provided better results than previous models but still struggled in capturing high level interaction. Later, Graph Neural Networks (GNN) became prominent in this field due to their ability in capturing intricate relationships between multiple siRNA-mRNA pairs through message passing. Models such as GNN4siRNA (La Rosa et al., 2022) and siRNADiscovery (Long et al., 2024) have since become state-of-the-art GNN models. GNNs require large and diverse data which is difficult to obtain in this domain and cannot be efficiently parallelized due to complex dependencies inherent in graph structures. Moreover, graph-based models tend to lose key structural details when handling imbalanced data, leading to over-smoothing (Keriven, 2022) or signal dilution, resulting in degraded performance.

To overcome these challenges, we introduce SiaRNA, a deep learning model that integrates a Siamese Neural Network (SNN) (Bromley et al., 1993) with bi-directional cross-attention (Vaswani et al., 2017) mechanism and thermodynamic feature integration for siRNA efficacy prediction. A Siamese Neural Network is a type of deep learning model that is good at understanding the similarity or compatibility between pairs of inputs by projecting them into a shared embedding space. The cross-attention mechanism is applied to capture the context-dependent relationships within sequential data. The feature representations of siRNA and mRNA independently processed before projecting them into a shared embedding space, enabling the model to learn biologically meaningful relationships between interacting molecules. In parallel, a bidirectional cross-attention module analyzes the nucleotide sequences of both siRNA and mRNA, allowing each sequence to identify and focus on the most relevant regions of its binding partner. This interaction-aware mechanism captures contextual dependencies that are difficult to model using conventional feature-based approaches alone. The outputs of these modules are further combined with thermodynamic stability features, which provide information about duplex formation and strand selection during RNA interference. By integrating feature-based learning, sequence-level interaction modelling and thermodynamic information within a unified architecture, SiaRNA provides a comprehensive framework for predicting siRNA efficacy. The model is trained and evaluated exclusively on experimentally validated 21-nucleotide siRNAs, ensuring consistency between biological observations and computational inputs.

## 2 Results

### 2.1 Model training

All experiments were conducted on a NVIDIA A100 GPU, which provided the computational capacity necessary to train the model on high-dimensional biological sequences. From the total 2,816 siRNA-mRNA pairs in Dataset_HUVK, 1971 were used for training, 423 for validation, and 422 for testing.

The model was trained using the Adam optimizer, chosen for its effective convergence. Gradient clipping was used to ensure numeric stability while training. This technique mitigates exploding gradients which is a common source of instability in deep networks. In addition, layer normalization was used to stabilize activation across layers, which improves training stability. This mechanism guarantees that the model’s weights are updated smoothly, leading to more stable convergence.

### 2.2 Hyperparameter configuration

The model’s training was controlled by a set of precise hyper parameters, summarized in Table 1. These parameters define the dimensions of all layers, from the initial high-dimensional projection of input features to the output dimensions of the shared Siamese embedder. They also specify the number of heads used in the cross-attention module and the layer sizes within the fusion layer and the regression Multi-Layer Perceptron (MLP).

**TABLE 1:** Summary of model hyper parameters.

Category	Hyperparameter	Value
Data and features	Max siRNA length	21
	Max mRNA length	9,756
	mRNA slice length	57
Model architecture	SNN projection dimension	256
	Hidden dimension	64
	Embedding dimension	32
	Attention heads	4
	Fusion dimension	128
	MLP dimension	64
Training	Learning rate	1e-3
	Epochs	20
	Batch size	32
	Gradient clip norm	1.0
Loss function	Lambda metric	0.1
	Contrastive margin	1.0

Table 1 lists the key training parameters and model parameters along with their corresponding values used during model development.

### 2.3 Performance on dataset_HUVK

To test the model, we benchmarked its performance on Dataset_HUVK against siRNADiscovery, using 422 siRNA-mRNA pairs (15% of the total dataset). The comparison validation metrics are presented in Table 2. The results demonstrate that SiaRNA achieves performance that is highly competitive and on par with the current benchmarks. Among the compared models, i-Score exhibited the weakest performance, having PCC, SPCC and MSE of 0.602, 0.623 and 0.064 respectively.

**TABLE 2:** Validation results on dataset_HUVK.

	siRNADiscovery			SiaRNA		
Metric	10-Fold split	Transcript split	CDHit split	10-Fold split	Transcript split	CDHit split
MSE	0.020	0.041	0.032	0.021	0.044	0.023
PCC	0.770	0.673	0.548	0.769	0.685	0.58
SPCC	0.771	0.692	0.591	0.775	0.717	0.618
AUC	0.874	0.846	0.806	0.881	0.86	0.824

Table 2 reports the validation metrics obtained on Dataset_HUVK, demonstrating the predictive performance of the proposed architecture. The best results are presented in bold.

### 2.4 Performance on the simone dataset

**FIGURE 1:**
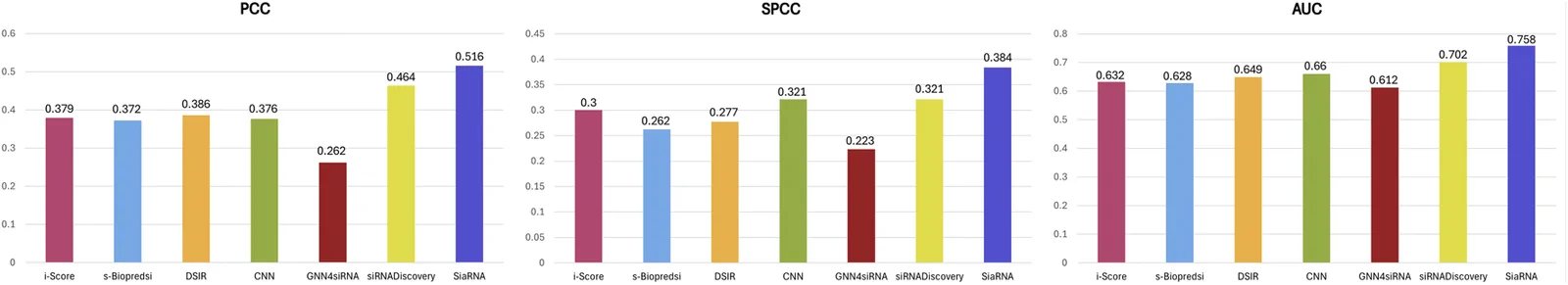
Comparative performance of models on Simone dataset.

To further assess the robustness and generalization of SiaRNA, it was evaluated on the independent external Simone dataset, which contained 322 siRNA-mRNA pairs. The results show that SiaRNA demonstrates strong predictive capability and achieves consistently higher performance across the evaluation metrics. SiaRNA achieves a PCC of 0.5163, SPCC of 0.3849 and AUC of 0.7586, compared to siRNADiscovery’s PCC of 0.464, SPCC of 0.32 and AUC of 0.702. As seen in Figure 1, SiaRNA outperforms other state-of-the-art models on the Simone dataset. These findings suggest that SiaRNA provides improved generalization on unseen data and effectively addresses some of the limitations observed in previous architectures.

Figure 1 represents three graphs comparing the performance of models against three important evaluation metrics, namely, Pearson correlation coefficient (PCC), Area under the ROC curve (AUC), and Spearman correlation coefficient (SPCC). For each graph, the x-axis represents the models, while its y-axis represents the corresponding evaluation metric score. All three graphs compare SiaRNA to existing state-of-the-art models and demonstrate overall improvements in predictive accuracy and generalization on the external Simone dataset. The values are given in Supplementary data Supplementary Table S1.

#### 2.4.1 Statistical significance of the model performance

Statistical significance testing on the Simone dataset showed that SiaRNA achieved a modest improvement in PCC compared with siRNADiscovery; however, this difference did not reach statistical significance (paired t-test, p = 0.074). In contrast, SiaRNA demonstrated a statistically significant improvement in SPCC over siRNADiscovery (paired t-test, p = 0.0018), accompanied by a large effect size (Cohen’s d = 1.38), indicating enhanced preservation of the relative ranking of siRNA efficacies. Furthermore, SiaRNA significantly reduced prediction error, as measured by MSE, relative to siRNADiscovery (paired t-test, p = 0.002), with a large effect size (|Cohen’s d| = 1.36). Detailed statistical results are provided in Table 3.

**TABLE 3:** Statistical testing on the simone dataset.

Metrics	Mean difference	Paired t-test t (9)		Wilcoxon W		Effect size Cohen’s d	95% confidence interval (CI)
		Value	p	Value	p		
PCC	0.0166	2.022	0.0739	9.0	0.0645	0.64	[-0.0020, 0.0352]
SPCC	0.0612	4.362	0.0018	3.0	0.0098	1.38	[0.0295, 0.0930]
MSE	−0.0043	−4.288	0.0020	1.0	0.0039	−1.36	[-0.0066, −0.0021]

Table 3 shows the statistical test results for the comparison between siRNADiscovery and SiaRNA on the Simone Dataset.

#### 2.4.2 Performance on Independent Datasets

For the Taka dataset, SiaRNA exhibited predictive performance comparable to siRNADiscovery, with no statistically significant differences observed for PCC, SPCC, or MSE. In contrast, on the Reynolds dataset, SiaRNA achieved significantly higher PCC and SPCC values than siRNADiscovery, indicating improved predictive generalization and ranking performance. The results are given in Table 4.

**TABLE 4:** Testing on independent datasets.

Metrics	Taka		Reynolds	
	SiaRNA	siRNADiscovery	SiaRNA	siRNADiscovery
PCC	0.6021	0.6016	0.208	−0.7664
SPCC	0.5992	0.5990	0.223	−0.7346
AUC	0.7858	0.8038	0.622	0.0361

Table 4 shows the testing results on Independent Datasets.

Statistical comparison between SiaRNA and siRNADiscovery on the Taka dataset showed no significant differences in PCC, SPCC, or MSE (all p > 0.05). The observed effect sizes were small, and the corresponding confidence intervals included zero, suggesting that the two models’ predictive performance was comparable on this dataset, as shown in Table 5.

**TABLE 5:** Statistical testing on the taka dataset.

Metrics	Mean difference	Paired t-test t (9)		Wilcoxon W		Effect size Cohen’s d	95% confidence interval (CI)
		Value	p	Value	p		
PCC	−0.0186	−1.714	0.1206	12.0	0.1308	−0.54	[-0.0433, 0.0059]
SPCC	−0.0184	−1.647	0.1337	12.0	0.1308	−0.52	[-0.0438, 0.0068]
MSE	−0.0018	−0.1775	0.8630	27.0	1.00	−0.05	[-0.0258, 0.0220]

Table 5 shows the statistical test results for comparison between siRNADiscovery and SiaRNA on the Taka Dataset.

On the Reynolds dataset, statistical testing demonstrated that SiaRNA significantly outperformed siRNADiscovery in both PCC and SPCC (paired t-test and Wilcoxon test, p < 0.01). These improvements were associated with very large effect sizes, indicating a substantial advantage of SiaRNA over siRNADiscovery in capturing and ranking siRNA efficacy patterns. Although SiaRNA achieved a lower MSE, this difference was not statistically significant. The detailed statistical results are presented in Table 6.

**TABLE 6:** Statistical testing on the Reynolds dataset.

Metrics	Mean difference	Paired t-test t (9)		Wilcoxon W		Effect size Cohen’s d	95% confidence interval (CI)
		Value	p	Value	p		
PCC	0.8856	29.335	0.00	0.00	0.0019	9.27	[0.8173, 0.9539]
SPCC	0.8026	25.267	0.00	0.00	0.0019	7.99	[0.7307, 0.8744]
MSE	−0.0667	−1.269	0.2360	14.0	0.1933	−0.40	[-0.1855, 0.0521]

Table 6 shows the statistical test results for comparison between siRNADiscovery and SiaRNA on the Reynolds Dataset.

### 2.5 Ablation study

**FIGURE 2:**
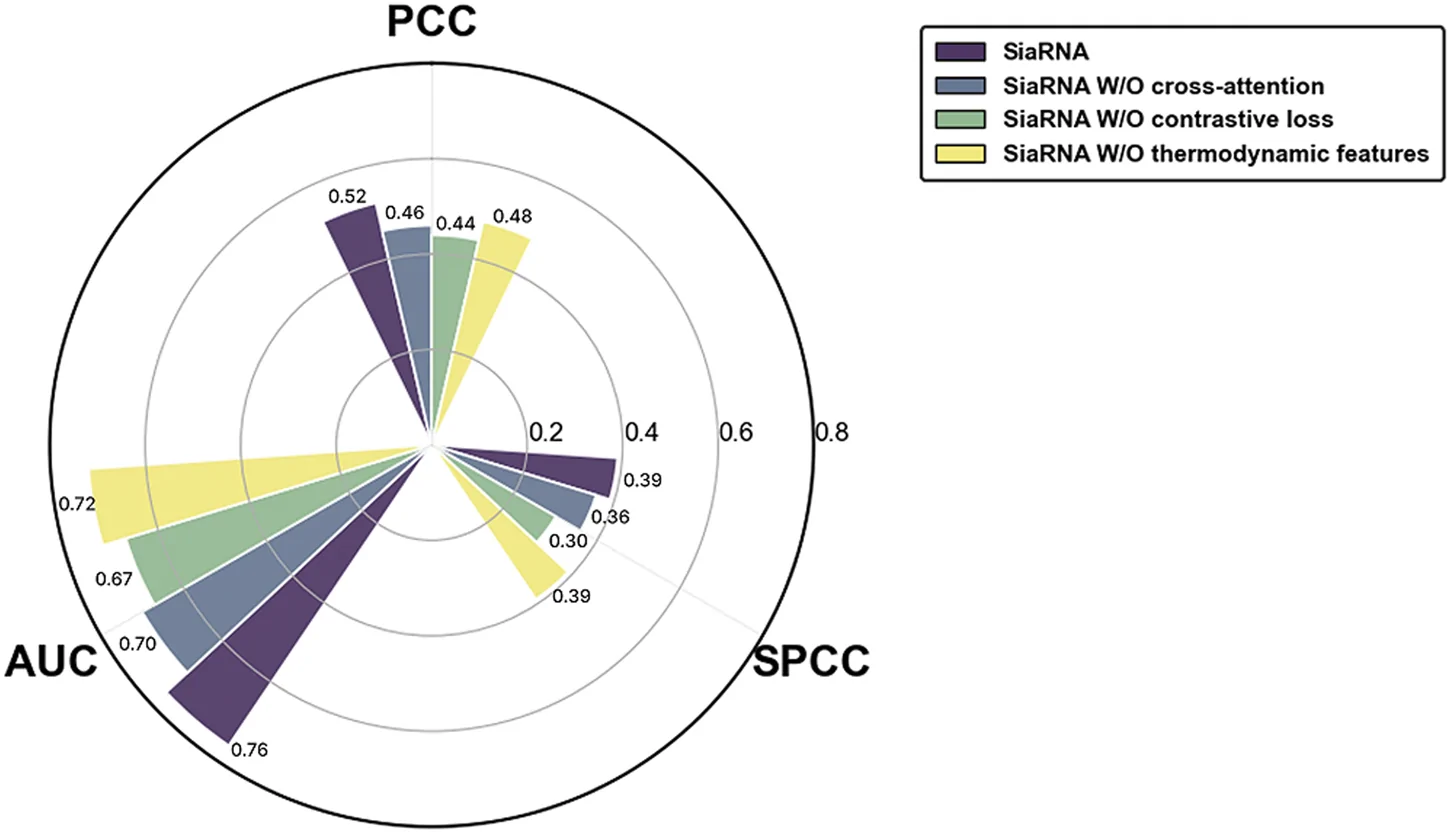
Comparison of ablated variants.

An ablation study was conducted as a means to investigate the individual contribution of key model components separately. The key components considered were the bidirectional cross attentional module, thermodynamic features and contrastive loss. The ablation of these model components was chosen because they represent the conceptually and functionally distinct elements in the architecture. The observed results of the ablation study are visualized in Figure 2.

Figure 2 shows ablation experiments done to validate the contribution of key modules and components of the proposed model architecture. In this, “W/O” stands for “without”, implying that certain modules, such as cross-attention, thermodynamic features, or contrastive loss, were removed to test their contribution individually.

The bidirectional cross-attention module is a core structural component that models the contextual dependencies between the mRNA and siRNA sequences. Evaluating its removal helps determine how much of the model’s predictive power comes from explicit sequence-level interaction learning. The thermodynamic feature module acts as an independent source of information derived from biophysical principles. By removing it, we investigate the degree to which the thermodynamic properties of the siRNA-mRNA pairs complement the learned features from the SNN and bidirectional cross-attention modules. Finally, the contrastive loss was ablated because it represents a novel addition to the learning objective, intended to enforce alignment between the siRNA and mRNA embeddings in the shared latent space. The removal of this component shows precisely its contribution in promoting generalization.

The effects of the handcrafted features were also investigated on model performance for each category mentioned in section 3.3.1. This ablation study showed that sequence composition and encoding-based features had the most effect on the model performance. The metrics for this ablation study are outlined in Supplementary Data Supplementary Tables S2, S3.

### 2.6 Case study

To assess the practical applicability of SiaRNA beyond benchmark datasets, a case study was conducted using Patisiran (Urits et al., 2020), a FDA-approved siRNA therapeutic. Patisiran targets the transthyretin (TTR) gene and has demonstrated clinical efficacy in the treatment of hereditary transthyretin-mediated amyloidosis. For this analysis, the validated Patisiran siRNA sequence and its target TTR mRNA transcript were provided as inputs to the model. The target mRNA aligned FASTA sequence (Gene Sequence ID: NM_000371.4) was downloaded from NCBI BLAST, annotated as *Homo sapiens* transthyretin (TTR), mRNA (Sequence ID: NP_000362.1). The image of the screen with the information is provided in Supplementary Figure S1.

SiaRNA predicted an efficacy score of 0.7134 for this validated siRNA-mRNA interaction, indicating a high likelihood of effective target silencing. The successful prediction of a high efficacy score for a clinically approved therapeutic suggests that the model can capture biologically meaningful determinants of siRNA activity. This case study provides additional evidence that SiaRNA may serve as a useful tool for prioritizing candidate siRNAs and supporting RNAi-based therapeutic design.

## 3 Methodology

### 3.1 Overview

SiaRNA is designed to capture thermodynamic, compositional, and contextual dependencies between siRNA and its target mRNA. Specifically, the model first processes a set of handcrafted input features using a Siamese Neural Network. In parallel, the raw nucleotide sequences of siRNA and the corresponding mRNA region are numerically encoded based on nucleotide identity and passed through a shared bi-directional cross attention mechanism.

The outputs from the feature-based and attention-based modules are then fused with thermodynamic features (Xia et al., 1998) and passed through fully connected layers to predict the final efficacy. The hybrid design enables the model to leverage both biologically interpretable features and to learn deep representations, achieving a comprehensive understanding of siRNA-mRNA interactions.

### 3.2 Data collection and characteristics

To train the model, 2,816 unique siRNA-mRNA pairs and their respective efficacies from Massimo et al (La Rosa et al., 2022) are used, which were originally derived from the studies of Huesken et al. (2005), Harborth et al. (2003), Ui-Tei et al. (2004), Vickers et al. (2003), and Khvorova et al. (2003) and presented in Table 7. This combined dataset is referred to as Dataset_HUVK. Further, the Simone dataset (Sciabola et al., 2013), consisting of 322 unique siRNA-mRNA pairs is used to test the model. A cell line is a population of cells derived from a specific tissue. They provide controlled biological environments for evaluating siRNA-mediated gene silencing and are widely used to assess the efficacy of RNA interference experiments. Dataset_HUVK encompasses cell lines H1299, HeLa, T24 and HEK293 and Simone encompasses Hep3B.

**TABLE 7:** Sources of dataset.

Dataset	Sources	siRNA	mRNA	Cell line
Dataset_HUVK	Huesken	2,431	34	H1299
	Harborth	44	1	HeLa
	Ui-tie	53	3	HeLa
	Vickers	76	2	T24
	Khovorova	14	1	HEK293
Simone	Simone	322	6	Hep3B

Table 7 summarizes the datasets used in this study, including their original sources, the number of siRNAs and target mRNAs, and the corresponding cell lines in which the efficacy measurements were experimentally obtained.

A 10-fold validation strategy is employed, splitting Dataset_HUVK (2,816 siRNA-mRNA pairs) in a 70:15:15 ratio into training (1971 siRNA-mRNA pairs), validation (423 siRNA-mRNA pairs) and test (422 siRNA-mRNA pairs) splits. The data is split ten times, and each time using different random seeds to generate ten distinct folds of data, which reduces sampling bias and ensures reliable evaluation of performance. The unique siRNAs and mRNAs count is presented in the Supplementary Data Supplementary Table S5.

Besides 10-fold validation, two additional data-splitting strategies were employed to evaluate model generalization under different levels of sequence similarity. For the transcript-level split, unique mRNA transcripts were partitioned into training, development, and test sets using a random seed of 42, ensuring that all siRNA samples associated with a given transcript were assigned exclusively to a single subset. This resulted in 2,006 training samples (27 transcripts), 540 development samples (9 transcripts), and 270 test samples (5 transcripts). For the homology-aware split, mRNA sequences were clustered using CD-HIT-EST at an 80% sequence identity threshold. The 41 unique mRNA transcripts were grouped into 40 clusters, and entire clusters were assigned to the training, development, or test set to prevent homologous sequences from appearing across multiple partitions. This yielded 1,968 training samples (24 unique mRNAs), 423 development samples (7 unique mRNAs), and 425 test samples (10 unique mRNAs), with no cluster leakage detected between subsets.

### 3.3 Feature engineering

The proposed framework utilizes two distinct input streams - one for the feature-engineered representations through the Siamese Neural Network (SNN) block and another for sequence-based representations for the Bidirectional Cross-Attention block.

#### 3.3.1 Feature inputs for siamese neural network

For the SNN component, comprehensive feature vectors are constructed from the sequences of siRNA and mRNA.

##### 3.3.1.1 Sequence composition and encoding

- One-Hot Encoding: To capture positional nucleotide identity, both the siRNA and mRNA sequences are encoded using binary one-hot encoding scheme with each base (N, A, G, C, U/T) being assigned a unique four-dimensional vector: N=<0,0,0,0>, A=<1,0,0,0>, C=<0,1,0,0>, G=<0,0,1,0>, U/T=<0,0,0,1> (Zeng et al., 2016).
- Nucleotide Frequencies (siRNA Only): To quantify the influence of short sequence motifs on siRNA function, the frequency of short segments (k-mers) is calculated for the sequences. Previous work shows that k-mers significantly influence knockdown efficacy (Vert et al., 2006). For example, dimer and trimer compositions have emerged as strong predictors of potency in machine learning models (Wang et al., 2010) and specific motifs such as UCC or A/U-rich regions - are statistically associated with enhanced silencing efficacy (Reynolds et al., 2004). The calculated frequencies are: 1-mer (A,U,C,G) with 4 motifs, 2-mer (e.g., AU, CG, etc.) with 16 possible motifs, 3-mer (e.g., AUC, UCG, etc.) with 64 possible motifs, 4-mer (e.g., AUCG, UCGA, etc.) with 256 possible motifs and 5-mer (e.g., AUCGA, UCGUA, etc.) with 1,024 possible motifs
- G/C Percentages: GC content for both the siRNA guide strand and the mRNA target sequence is computed. GC content is a critical factor influencing the stability of the siRNA-mRNA binding. Low GC content can lead to weak, non-specific binding, while excessively high GC content can hinder the essential unwinding of the siRNA duplex by the RISC complex, thus impeding efficacy (Reynolds et al., 2004; Liu et al., 2013).

##### 3.3.1.2 Interaction and rule-based features

- RNA-AGO2 Interaction Score: The score represents a quantitative measure of the RNA-protein interaction (Birmingham et al., 2006) between the RNA (siRNA or mRNA) transcript and the Argonaute 2 (AgO2) protein (Sasaki and Tomari, 2012). The score is computed using ZHMolGraph (Liu et al., 2025), a framework designed to capture the structural and chemical interactions between RNAs and proteins. The computed score represents the RISC loading potential of siRNA guide strand, which is a key biological feature for predicting efficacy.
- Rule Codes (siRNA Only): To encode the established knowledge that certain nucleotides at specific positions enhance or impair siRNA function, simplified rule codes are applied to the siRNA guide strand, based on the findings of He et al (Sciabola et al., 2013). The original preference scores (1 for enhancing, −1 for reducing, 0 for no preference) are converted into unique-three dimensional binary vectors: 1=<0,0,1>, 0=<0,1,0>, −1=<1,0,0>.

##### 3.3.1.3 Structural features

- Base Pairing Probabilities: The nucleotide base pairing within the individual strands (siRNA and mRNA) drives the formation of their secondary structures. This folding is biologically relevant because, within the cellular environment, both RNA transcripts exist primarily as folded species, not as linear strands. Both canonical (A-U, C-G) and non-canonical (G-U) interactions are considered. The RNAfold tool from ViennaRNA (Lorenz et al., 2011) package is used to obtain the base-pairing probability matrix for the individual siRNA and mRNA sequences. Due to the size and sparsity of these base-pairing probability matrices, truncated Singular Value Decomposition (SVD) is applied for dimensionality reduction, yielding compact representations of six dimensions for siRNA and one hundred dimensions for mRNA, which are subsequently used as model features.

#### 3.3.2 Feature inputs for bidirectional cross attention

For the bi-directional cross attention module, siRNA and mRNA sequences are represented as tokenized nucleotide indices (Ji et al., 2021). Each siRNA (21-nt) is fully encoded, while for the mRNA, a fixed 57-nt (Qiu et al., 2005) slice centered around the siRNA binding site is extracted. The mRNA slice has 28-nt upstream and 29-nt downstream of the target binding site. If the extracted mRNA sequence is shorter than 57-nt, suitable padding is applied to ensure compatible input length. Each nucleotide within these sequences is then numerically encoded into integer indices using a predefined mapping as: adenine (A) to 0, thymine (T) and uracil (U) to 1, guanine (G) to 2, cytosine (C) to 3 and ambiguous bases (N) to 4. This encoding is implemented by converting each nucleotide into its corresponding integer index and forming a tensor, which acts as the input to the attention mechanism. This representation allows the siRNA and target mRNA to be in uniform and computationally efficient format to analyze the contextual dependencies between them.

### 3.4 Architectural framework and core components

**FIGURE 3:**
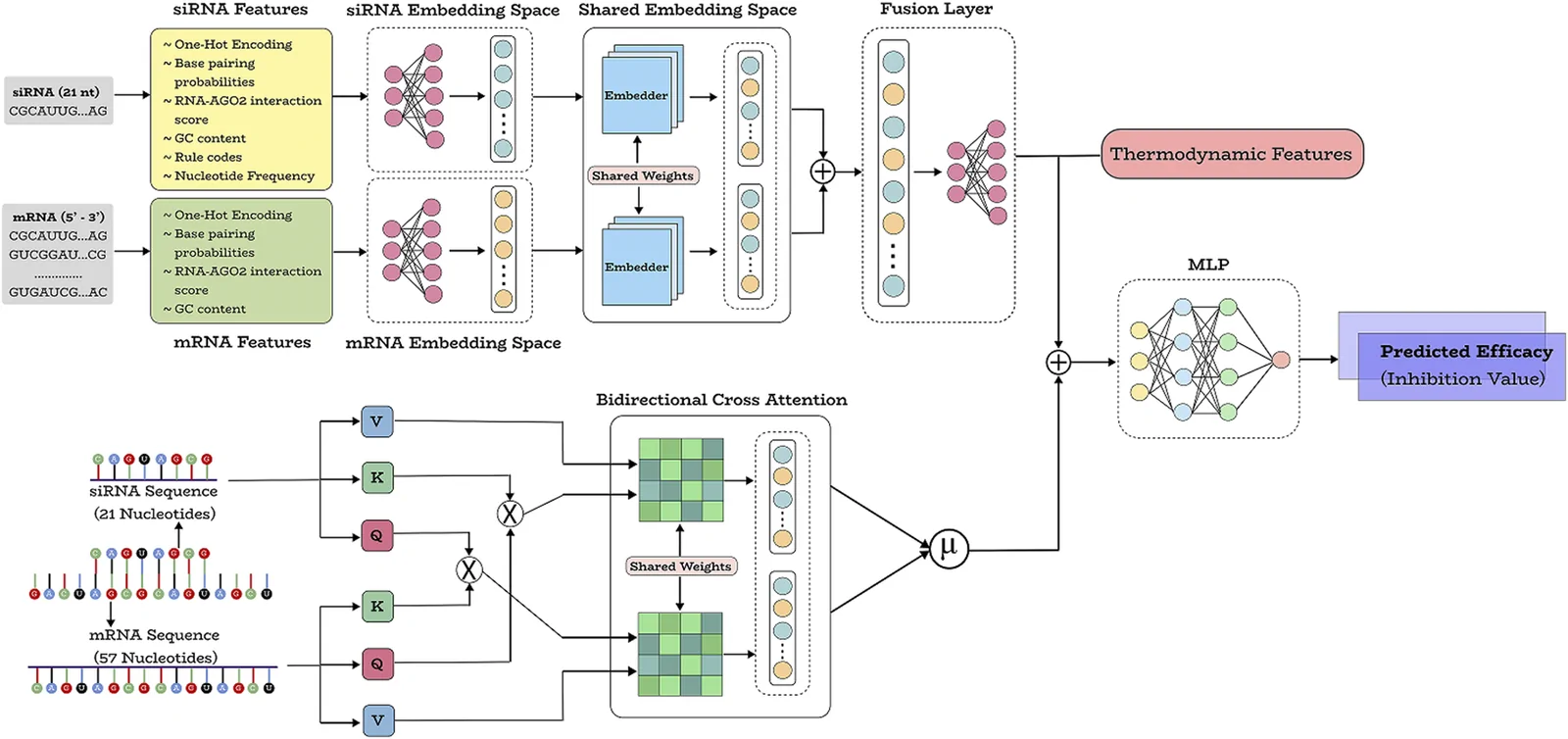
Architecture Diagram of SiaRNA. Figure 3 illustrates the end-to-end framework comprising two primary modules: a Siamese neural network for extracting pairwise characteristics and a bidirectional cross-attention module for capturing sequence-level relationships. The outputs from both modules are concatenated with thermodynamics features and passed through a multilayer perceptron (MLP) for final efficacy prediction.

The proposed architecture of SiaRNA integrates sequence-derived, contextual and thermodynamic information in order to achieve accurate modelling of siRNA-mRNA interactions. As depicted in Figure 3, it consists of three key components: a Siamese Neural Network (SNN), a bidirectional cross-attention module and a thermodynamic-MLP component, where precomputed thermodynamic stability features are concatenated with the learned embeddings and processed through a MLP for final efficacy prediction.

#### 3.4.1 Siamese neural network

A Siamese Neural Network (SNN) is a specialized architecture that consists of two or more identical subnetworks, each sharing weights and parameters, designed to process pairs of inputs in parallel. While traditional neural networks predict specific outputs over individual inputs, SNNs are good at comparing features which makes them particularly effective for applications where the relationship or compatibility between two entities is to be studied.

##### 3.4.1.1 Linear projection layers

The linear projection layers provide the starting point for dealing with the raw handcrafted features of the siRNA and mRNA. These transform the features of different sizes and scales into fixed dimension vectors and project these vectors into the respective siRNA and mRNA embedding spaces. Independent processing of handcrafted features allows the model to learn more effectively by allowing the siRNA and mRNA to develop their own feature representations before interacting.

##### 3.4.1.2 Shared embedder

The shared embedder component processes the standardized siRNA and mRNA features using the same weights for both inputs, projecting them into a shared embedding space, thereby forcing the model to learn a common language. This allows the model to measure the compatibility, or “distance,” between the siRNA and mRNA in the shared space, a step essential for learning patterns that define a successful interaction.

##### 3.4.1.3 Fusion layer

The Fusion Layer is designed to concatenate the shared representations of the siRNA and mRNA feature vectors into a single, cohesive vector. The combined vector is then fed into a fully connected layer which models the complex, non-linear interactions between the siRNA-mRNA pairs. The goal of this component is to move beyond the individual characteristics and create a unified representation ( $h_{SNN}$ ) that captures the relational properties of the siRNA-mRNA pairs.

#### 3.4.2 Bi-directional cross attention for nucleotide-level interaction

The Bidirectional Cross-Attention mechanism is a key component of the model, designed to simulate the subtle yet dynamic interaction between the 21-nt siRNA and its target 57-nt mRNA slice. The task of this component, beyond simple matching of sequences, is to learn complex position-dependent dependencies that influence binding affinity. This mechanism operates as a bi-directional, two-way process, effectively modelling the interaction between the two sequences to determine their compatibility.

The attention mechanism provides the model with the ability to selectively concentrate on the most relevant parts of the input. It computes a weighted sum of input features, where the weights indicate the importance of each input element for the current output. Mathematically, given queries Q , keys K and values V , attention is calculated as:

$$Attention\left(Q,K,V\right)=\text{Softmax }\left(\frac{QK^{T}}{\sqrt{d_{k}}}\right)V$$

Here, $QK^{T}$ computes similarity scores between the query and keys, scaled by $\sqrt{d_{k}}$ (the dimension of the keys) for numerical stability. The softmax function normalizes these scores into attention weights, which are then used to compute a weighted sum of the values V . This process allows the model to prioritize relevant information dynamically for each step in the output generation.

In our model, the interaction is performed bi-directionally using shared weight matrices $\left(W_{Q},W_{K},W_{V}\right)$ for the linear projections of the query, key and value inputs. First, the input encoding of the mRNA slice $\left(X_{mRNA}\right)$ is projected to form the query $\left(Q_{mRNA}=X_{mRNA}W_{Q}\right)$ . Simultaneously, the siRNA encoding $\left(X_{siRNA}\right)$ is projected to form the key $\left(K_{siRNA}=X_{siRNA}W_{K}\right)$ and value $\left(V_{siRNA}=X_{siRNA}W_{V}\right)$ . For each nucleotide in the mRNA, the attention mechanism scans the entire siRNA, resulting in a contextualized representation of mRNA $\left(H_{mRNA}\right)$ informed by the most salient features of its binding partner.

$$H_{mRNA}=\text{Softmax }\left(\frac{Q_{mRNA}K_{siRNA}^{T}}{\sqrt{d_{k}}}\right)\left(V_{siRNA}\right)$$

Then, the roles are reversed, but the same shared projection weights $\left(W_{Q},W_{K},W_{V}\right)$ are utilized. The siRNA sequence forms the query $\left(Q_{siRNA}=X_{siRNA}W_{Q}\right)$ , while the mRNA sequence forms the key $\left(K_{mRNA}=X_{mRNA}W_{K}\right)$ and value $\left(V_{mRNA}=X_{mRNA}W_{V}\right)$ . This creates a contextualized representation of the siRNA $\left(H_{siRNA}\right)$ , with its features now structurally informed by the mRNA target region.

$$H_{siRNA}=\left(\frac{Q_{siRNA}K_{mRNA}^{T}}{\sqrt{d_{k}}}\right)\left(V_{mRNA}\right)$$

Finally, a single output vector is acquired by computing the element-wise average of the two attention output matrices.

$$h_{CA}=\frac{H_{mRNA}+H_{siRNA}}{2}$$

The shared bidirectional cross-attention mechanism captures the reciprocal nature of siRNA-mRNA interactions. It provides for the two sequences to be projected onto a common embedding space through the use of shared query, key, and value projections for symmetrical information exchange. siRNA-mRNA pairing is not a one-way recognition process but a mutually aligned biologically meaningful interaction. Each sequence refines its internal representation based on cues emanating from its partner through the bidirectional cross-attention mechanism, effectively mirroring the mutual recognition process inherent in RNA-RNA binding. This allows the model to learn higher-order relational dependencies and generalize to diverse sequence contexts. The bidirectional cross-attention comparison of shared and separate weights is presented in the Supplementary data Supplementary Table S6.

#### 3.4.3 Thermodynamics features and MLP

The thermodynamic stability profile of the siRNA duplex is one of the key determinants of its silencing efficiency. The energy asymmetry between the two ends of the duplex determines which strand is preferentially loaded into the RNA-induced silencing complex (RISC), thereby affecting the overall gene-silencing outcome. To quantitatively assess this property, feature engineering is performed to compute multiple thermodynamic parameters like the Watson- Crick pair free energy (ΔG) between adjacent nucleotide pairs, the total duplex free energy and the thermodynamic asymmetry between the 5′ and 3′ ends (Lu et al., 2006). The thermodynamic features were calculated using the methodologies described by Xia et al. (1998), Consecutive nucleotide pairs were considered and assigned values for each pair from this study. The free energy (ΔG) serves as a measure of thermodynamic favorability, where lower values generally indicate more stable and energetically favorable interactions. These features which are derived from established biophysical and thermodynamic models represent the duplex’s energy landscape and its contribution to efficacy prediction.

To perform the efficacy prediction, the feature representations obtained from the cross-attention module and SNN are concatenated with the pre-computed thermodynamic feature vector ( $h_{SNN}$ ). This gives a unified feature vector ( $h_{concat}$ ) that has sequential, structural and biophysical information:

$$h_{concat}=Concat\left(h_{SNN},h_{CA},h_{thermo}\right)$$

The comprehensive and high-dimensional feature vector is then passed into a MLP (Multi-layer perceptron) which acts as a regression head (Qin et al., 2022). The MLP captures complex, non-linear relationships within the integrated features. Its output layer then transforms the learned representation into a single continuous value, representing the model’s predicted siRNA-mRNA efficacy score.

### 3.5 Loss functions

The model is trained using a hybrid objective that integrates a regression loss for efficacy prediction and contrastive loss to align the latent representations of the siRNA and mRNA embeddings.

The primary objective is the Mean Squared Error (MSE) (Huesken et al., 2005) between the predicted and experimentally measured efficacies to perform regression:

$$L_{reg}=\frac{1}{N}\sum_{i=1}^{N}{\left(y_{i}-\hat{y_{i}}\right)}^{2}$$

To encourage the model to differentiate effective from ineffective interactions, contrastive loss (Hadsell et al., 2006) between the siRNA and mRNA embeddings is obtained from the shared embedding space:

$$L_{con}=\frac{1}{N}\sum_{i=1}^{N}\left[y_{i}d_{i}^{2}+\left(1-y_{i}\right)\;\max{\left(0,m-d_{i}\right)}^{2}\right]$$

where $d_{i}={\left\|z_{m,i}-z_{s,i}\right\|}_{2}$ denotes the Euclidean distance between the paired embeddings, $y_{i\;}$ is a binary label derived from efficacy (1 for efficacy >0.7, 0 otherwise (Qureshi et al., 2013), and *m* is contrastive margin.

The final loss is a weighted sum of the two objectives:

$$L=L_{reg}+\lambda L_{con}$$

where λ controls the contribution of the contrastive term.

The joint optimization encourages the network to produce both accurate efficacy predictions and semantically align the siRNA-mRNA embeddings, improving generalization and learning meaningful relationships between interacting molecules.

### 3.6 Evaluation metrics

The performance of the model is evaluated using both regression and classification-based metrics.

For regression, the metrics reported are:

Mean Squared Error (MSE) (La Rosa et al., 2022): It is the mean squared difference between predicted and the experimentally validated efficacy values. It helps us understand how close the model’s predictions are to the truth values.

Pearson Correlation Coefficient (PCC): It is used to measure the strength and direction of the linear relationship between the predicted efficacy values and the actual efficacy values.

Spearman Correlation Coefficient (SPCC) (Long et al., 2024): It is used to measure the monotonic relationship between predicted values and ground truth values independent of scale.

For classification-based evaluation, siRNAs with efficacy >0.7 are considered effective, and the rest are considered as ineffective. Using this binary threshold, Area Under Receiver Operating Characteristic Curve (AUC) is computed as a metric, showing the model’s ability to distinguish functional from non-functional siRNAs.

Using both regression and classification-based metrics is important as knockdown efficacy is inherently continuous, yet it is biologically interpreted in discrete terms such as effective or ineffective. This combined evaluation captures both the quantitative prediction accuracy and the functional relevance of the model in the biological context of RNAi.

#### 3.6.1 Statistical analysis

To assess whether the performance differences between SiaRNA and siRNADiscovery were statistically significant, both models were trained and evaluated using the same experimental protocol. The HUVK dataset was partitioned ten times into training and validation subsets using ten distinct random seeds. While the same set of experimentally validated siRNA–mRNA pairs was repartitioned across runs, each partition represented an independent realization of the train-validation split. Both SiaRNA and siRNADiscovery were trained independently from random initialization on each partition and subsequently evaluated on the same external Simone dataset, which remained unchanged throughout all experiments. This procedure yielded 10 pairs of performance values (PCC, SPCC, MSE, and AUC) for each model.

The statistical unit of analysis was the aggregated performance metric from each experimental run rather than the individual siRNA–mRNA predictions. Treating individual predictions as independent observations would violate the independence assumption because predictions within a run originate from the same trained model and because the training data are repeatedly partitioned from the same HUVK dataset. Instead, statistical comparisons were performed on the ten paired aggregate metrics, with each pair corresponding to the performance of SiaRNA and siRNADiscovery trained under the same data partition and evaluated on the identical external Simone dataset.

The paired observations were compared using a paired Student’s t-test as the primary statistical test. To validate the results without assuming normality of the paired differences, the Wilcoxon signed-rank test was additionally performed. The magnitude of the observed differences was quantified using Cohen’s d, while 95% confidence intervals (CI) were calculated to estimate the precision of the mean paired differences. (Rainio et al., 2024).

## 4 Discussion

In this section, the key design decisions underlying our model were reflected and their implications for siRNA-mRNA efficacy prediction were interpreted.

### 4.1 Use of 21-nucleotide siRNAs

Many earlier deep learning approaches have adopted 19-nucleotide (nt) siRNA sequences, largely to maintain consistency with early datasets and simplify preprocessing. Some recent deep learning approaches use efficacy labels derived from 21-nt sequences to train models on truncated 19-nt sequences (Bai et al., 2024), introducing a fundamental inconsistency in the input-output mapping. In contrast, the experimentally validated Huesken dataset, which we employ for training and validation, provides efficacy measurements for 21-nt sequences. The 21-nt length includes the characteristic two-nucleotide 3′ overhangs that are crucial for recognition and loading of the siRNA by the RNA-induced silencing complex (RISC). Experimental evidence suggests that 21-nt siRNAs exhibit superior stability and silencing efficiency compared to their 19-nt counterparts, as the 3′ overhang facilitates AGO2-mediated strand loading and target recognition (Martinez et al., 2002). This fuller representation allows deep learning models to learn more effective features, often leading to superior performance when trained on the 21-nt sequences compared to the truncated 19-nt version (Supplementary data Supplementary Table S4). This biologically grounded choice ensures alignment with experimental conditions under which efficacy labels were derived, thereby improving model validity.

### 4.2 Pairwise modelling of siRNA-mRNA interactions

Most graph-based RNA interference models represent mRNAs as central hubs connected to multiple siRNAs, forming star-like topologies. While this approach captures network-level relationships, it can obscure pair-specific effects and limit inference on new siRNA-mRNA combinations. In contrast, our model treats each siRNA-mRNA interaction as a distinct pair, enabling it to learn the specific binding patterns that arise from their joint sequence context (Birmingham et al., 2006). This methodology directly reflects the molecular mechanism of RNA interference (RNAi), where the functional outcome depends on the compatibility between a single siRNA guide strand and its target mRNA sequence. As a result, our model is better suited for predicting the efficacy of novel siRNA-mRNA pairs not encountered during training, an essential feature for practical siRNA design. This can be observed from our model’s superior performance over graph based models like GNN4siRNA and siRNADiscovery as shown in Supplementary Data Supplementary Table S1.

#### 4.2.1 Shared embedding space for siRNA and mRNA

The Siamese Neural Network (SNN) architecture used in our model allows siRNA and mRNA to be encoded through parallel pathways before being projected into a shared latent representation. This design has an intuitive biological interpretation: if a particular siRNA effectively silences its target, their embeddings in this shared space will be closer together; conversely, pairs with weak or no silencing potential will be more distant. It encourages biologically meaningful alignment between siRNA and mRNA features and enhances the model’s capacity to learn cross-molecular compatibility. This shared embedding mechanism thus provides a means to quantify the “functional proximity” between siRNA and mRNA sequences beyond surface-level similarity.

### 4.3 Bidirectional cross-attention for mutual context modelling

The bidirectional cross-attention mechanism captures the reciprocal nature of siRNA-mRNA interactions. The use of shared query, key, and value projections ensures that the two sequences are represented in a common embedding space, facilitating symmetrical information exchange. This symmetry is biologically meaningful: siRNA-mRNA pairing is not a one-way recognition process but a mutual alignment. Through this bidirectional attention, each sequence refines its internal representation based on cues from its partner, effectively mirroring the mutual recognition process inherent in RNA-RNA binding. This mechanism enables the model to learn higher-order relational dependencies, allowing it to generalize to diverse sequence contexts.

Together, these components form a cohesive architecture that captures both the individual and interactive characteristics of siRNAs and their mRNA targets. The biologically grounded design choices - particularly the use of 21-nt sequences, pairwise modelling, shared embedding spaces, and bidirectional attention - collectively enhance generalization and predictive performance, offering a robust framework for siRNA efficacy prediction and future RNA-based therapeutic design.

## 5 Conclusion

The present work introduces SiaRNA, a deep learning framework for prediction of siRNA-mRNA efficacy, based on biologically aware feature engineering combined with a Siamese Neural Network and bidirectional cross-attention architecture. Our model leverages handcrafted sequence-derived features like nucleotide composition, GC content, base-pairing probabilities and nucleotide frequencies along with contextual sequence embedding. This allows the model to capture both intrinsic sequence properties and pairwise interaction dynamics. Encoding siRNA and mRNA sequences independently prior to projection into a common embedding space helps preserve and enrich their individual feature representations. The bidirectional cross-attention mechanism plays a major role in capturing mutual, context-dependent interactions which reflect the dynamic and reciprocal nature of RNA interference. The model is trained and validated on Dataset_HUVK and tested on the independent Simone dataset. SiaRNA yields an improvement in generalization and predictive performance over previous approaches.

Despite its strong predictive performance, SiaRNA has several limitations that present opportunities for future improvement. First, the current framework focuses exclusively on silencing efficacy and does not model off-target effects, which are a critical consideration in therapeutic siRNA design. Future work could address this limitation by incorporating off-target prediction modules, or multi-task learning frameworks that simultaneously predict efficacy and off-target risk. Second, the model does not account for factors influencing *in vivo* performance such as delivery mechanism and chemical modifications. Integrating experimental delivery data, modification-specific features, and other complementary biological information through multimodal learning approaches could improve the model’s translational relevance. Finally, certain handcrafted features, such as AGO2 interaction scores, rely on external computational tools, which may affect reproducibility and deployment efficiency. Future models could address this challenge by learning such representations directly from sequence data through end-to-end training, reducing dependence on external feature-generation pipelines.

SiaRNA provides a dependable framework for the design of therapeutic siRNAs, allowing for the identification of more accurate siRNA candidates for gene silencing applications. Beyond therapeutics, the combination of feature-driven and attention-based modelling can enable broader gene regulation research and RNA-targeted interventions. This offers insights into the sequence-structure-functional relationships which regulate RNA-RNA interactions. This paves the way for integration with chemically modified siRNAs and the development of generalized RNA-targeted design approaches in diverse biological contexts.

## Data Availability

The original contributions presented in the study are included in the article/Supplementary Material, further inquiries can be directed to the corresponding author.
