# Supplementary material for: SiaRNA: a siamese neural network with bidirectional cross-attention for pairwise siRNA-mRNA efficacy prediction
Source: Front Bioinform. 2026 Aug 5;6:1827877. doi: 10.3389/fbinf.2026.1827877 (PMC13488420; doi:10.3389/fbinf.2026.1827877)
Supplement: Supplementary file 1 [file Supplementaryfile1.docx]

**SUPPLEMENTARY DATA**

**Table S1:** Evaluation Metrics among different Models

| Model | PCC | SPCC | AUC |
| --- | --- | --- | --- |
| i-Score  s-Biopredsi | 0.379  0.372 | 0.300  0.262 | 0.632  0.628 |
| DSIR  CNN  GNN4SiRNA  SiRNADiscovery  SiaRNA | 0.386  0.376  0.262  0.464  **0.516** | 0.277  0.321  0.223  0.321  **0.384** | 0.649  0.660  0.612  0.702  **0.758** |
|  |  |  |  |

Table S1 provides evaluation metrics on the Simone dataset for previous SOTA models. These values correspond to the values in Figure 1.

**Table S2:** Comparison of Ablation Variants

| Features | PCC | SPCC | AUC |
| --- | --- | --- | --- |
| All  W/O Cross Attention  W/O Contrastive Loss  W/O Thermodynamic features | 0.52  0.46  0.42  0.48 | 0.39  0.36  0.30  0.39 | 0.76  0.70  0.67  0.72 |

Table S2 provides ablation study values for Figure 2 on the Simone dataset.

**Table S3:** Ablation study on the HUVK dataset.

| Features | PCC | SPCC | AUC |
| --- | --- | --- | --- |
| All | 0.769 | 0.775 | 00.881 |
| W/O Sequence Composition and Encoding | 0.565 | 0.58 | 0.794 |
| W/O Interaction and Rule-Based Features | 0.74 | 0.75 | 0.87 |
| W/O Structural features | 0.75 | 0.76 | 0.87 |

Table S3 provides ablation study results for the three feature categories on the HUVK dataset.

**Table S4:** Comparison of performance for 19nt vs 21nt.

|  | **siRNADiscovery** | | **SiaRNA** | |
| --- | --- | --- | --- | --- |
| **Metric** | **19nt** | **21nt** | **19nt** | **21nt** |
| **MSE** | 0.022 | **0.020** | 0.021 | 0.021 |
| **PCC** | 0.733 | **0.770** | 0.733 | 0.769 |
| **SPCC** | 0.734 | 0.771 | 0.731 | **0.775** |
| **AUC** | 0.864 | 0.874 | 0.860 | **0.881** |

Table S4 provides the metrics for siRNADiscovery and SiaRNA on Dataset_HUVK for 19-nt and 21-nt length siRNA sequences.

**Table S5:** Overlap between Dataset_HUVK and Simone.

| Metric | Dataset_HUVK | Simone | Shared |
| --- | --- | --- | --- |
| Unique siRNAs | 2816 | 322 | 0 |
| Unique mRNAs | 41 | 6 | 0 |
| Unique pairs | 2816 | 322 | 0 |

Table S5 shows the overlap between mRNAs and siRNAs of Dataset_HUVK and Simone.

**siRNA Sequence Similarity Analysis**

- 17 Simone siRNAs have the nearest HUVK siRNA at Hamming distance 6 (15/21 identical nts, 71.4% identity)
- 65 Simone siRNAs have the nearest HUVK siRNA at Hamming distance 7 (14/21 identical nts, 66.7% identity)
- 166 Simone siRNAs have the nearest HUVK siRNA at Hamming distance 8 (13/21 identical nts, 61.9% identity)
- 73 Simone siRNAs have the nearest HUVK siRNA at Hamming distance 9 (12/21 identical nts, 57.1% identity)
- 1 Simone siRNAs have the nearest HUVK siRNA at Hamming distance 10 (11/21 identical nts, 52.4% identity)

**Summary:**

- Total Simone siRNAs analyzed: 322
- Minimum observed distance: 6
- Maximum observed distance: 10
- Mean nearest-neighbor distance: 7.93

**Table S6:** Comparison of shared vs. Separate weight bi-directional cross-attention

| Metric | Shared Weights | Separate Weights |
| --- | --- | --- |
| PCC | **0.749** | 0.769 |
| SPCC | **0.756** | 0.775 |
| AUC | **0.873** | 0.881 |

Table S6 shows the metric comparison between using a shared weight bi-directional cross-attention module and a separate weight bi-directional cross-attention module

**Figure S1:** Case study

**Figure S1:** Case study information of the TTR gene. Figure S1 provides a detailed description of the gene used in the case study – TTR- and the mRNA sequence IDs.
